# Management of Femur and Tibial Leg Length Discrepancies With a Unilateral External Fixator Is Still Viable When More Advanced Techniques and Hardware Are Unavailable or Cost-Prohibitive

**DOI:** 10.7759/cureus.21010

**Published:** 2022-01-07

**Authors:** Neritan Borici, Ekene U Ezeokoli, Julian Ruci, Taulant Olldashi

**Affiliations:** 1 Orthopaedic Surgery, University of Medicine, European Hospital Villa Maria, Tirana, ALB; 2 Orthopaedic Surgery, Texas Children's Hospital, Houston, USA; 3 Orthopaedics, National Trauma Center, Tirana, ALB; 4 Orthopaedics, AMEOS Group Hospital, Bremen, DEU

**Keywords:** lld, congenital, bone loss, bone growth, bone lengthening, leg length discrepancy, circular external fixator, monolateral external fixator, unilateral external fixator, cost

## Abstract

Introduction

Leg length discrepancy (LLD) is an infrequent diagnosis, most commonly occurring congenitally in children and rarely in traumatic incidents in adults. Circumferential external fixators are considered the optimal treatment method, but can be very costly and are not always readily available in less developed nations. The unilateral external fixator predates the circumferential but is more easily available and accessible worldwide and less expensive. This study sought primarily to characterize treatment outcomes using a unilateral external fixator where more advanced forms of treatment for LLD are not available. Secondary objectives included the site of the discrepancy and comparison of etiologies.

Methods

Data were retrospectively reviewed from January 2010 to December 2017 on patients undergoing unilateral external fixation at our institution. Nineteen patients met the criteria, 14 with congenital LLD and five with lower leg bone loss from trauma. Patient demographics (including gender and age), initial presentation, physical examination findings, radiographic findings, and treatment were collected and saved in an electronic medical record.

Results

There were 19 cases of LLD overall, with 14 cases on the tibia and 5 on the femur. Three of the five femur cases occurred in the trauma subgroup. There were 15 cases of congenital LLD and five cases of traumatic LLD. The mean overall LLD was 3.9 cm (2.3-5.2). The mean follow-up until healing for the entire cohort was 10 months (5-22). Patients with congenital LLD were younger than those with traumatic LLD (10.2 years versus 22.5 years, p=0.000013), more likely to have a tibial discrepancy (p=0.034), and had a shorter time frame until full healing (7.6 months versus 19 months, p 0.00001). Patients with a tibial LLD were more likely to have a congenital etiology (p=0.0374) and had a shorter time until full healing compared to patients with a femur LLD (8.5 months versus 14 months, p=0.03541).

Conclusion

We conclude that bone lengthening utilizing the unilateral external fixator is a good method and is cost-effective for bone lengthening where more advanced techniques are not available or cost-prohibitive. It is simple, and patients and families can collaborate with the surgeon to get a good final result. Patients are generally satisfied and can ambulate well after healing. In a resource-limited environment with cost as a barrier, if used correctly and judiciously, the unilateral external fixator can yield good results.

## Introduction

Leg length discrepancy (LLD) is not a frequent clinical finding. Etiology is variable, but due to the inequality of lower limbs, limping is the most notable complaint. As a consequence, the pelvis loses equilibrium and its loss of horizontal positioning may cause deformities in the vertebral column, such as scoliosis [1]. Joint pain and movement restriction are often eventual sequelae if LLD is left untreated. To avoid the symptomology and development of other pathologies, LLD must be treated definitively as soon as possible.

When LLD is minimal, treatment is conservative, varying from physiotherapy procedures to modifications in shoe heels [2]. In larger discrepancies, complaints are frequent and surgical treatment is necessary. There have been many surgical methods used to equalize the lower extremities. When the physes are still open, controlling growth by temporarily blocking the contralateral physis is an efficient, minimally invasive method called epiphysiodesis [3]. With a larger discrepancy and an older patient, other methods should be considered [4]. Proper bone elongation via surgery to realign limbs is the gold standard [5].

Techniques of bone lengthening vary, and the hardware used for bone lengthening has advanced over time. The circular external fixator, unilateral external fixator, and axial internal fixator are the most frequently used for the treatment of LLD [5]. The basic principle behind these techniques is the same. They take advantage of osteogenesis by slow distraction after osteotomy of the indicated bone, known as callotasis. The bone-lengthening process involves the movement of bone fragments by distracting and compressing them periodically by 1 mm per day. In this manner, the micro-movements stimulate the bone faster for the osteointegration process [6].

Difficulties and complications include axial deviation, neurologic or vascular injury, delayed consolidation, non-union, and muscle contracture [7]. Circumferential external fixators are often used for the treatment of LLD due to their efficacy and accuracy for the correction of length and axial deviations. This combination of hardware materials, technique, and increased development of new technology has diminished complications and given good results, but it has increased the cost of treatment [8,9]. When patients have no bone angulation or bowing and only require axial lengthening, some studies have found that good results can be yielded by the meticulous application of the traditional unilateral external fixator [6].

This study primarily aims to characterize treatment outcomes through time to healing in LLD after unilateral external fixation in a population where more advanced methods of treatment are unavailable or deemed too costly. Secondary aims include identifying leg length discrepancy variations, bony sites of the lesion causing discrepancy, and comparison of etiologies.

## Materials and methods

With permission and exemption from the institutional review board, data on patients undergoing unilateral external fixation were reviewed retrospectively from January 2010 to December 2017. Nineteen patients met the criteria, 14 with congenital LLD and five with lower leg bone loss from trauma. Patient demographics (including gender and age), initial presentation, physical examination findings, radiographic findings, and treatment were collected and saved in an electronic medical record.

All patients diagnosed with LLD underwent an X-ray. After an X-ray to confirm, a computed tomography scan (CT, scout view) was performed, and the discrepancy was electronically measured via CT.

Surgical technique

**Figure 1:**
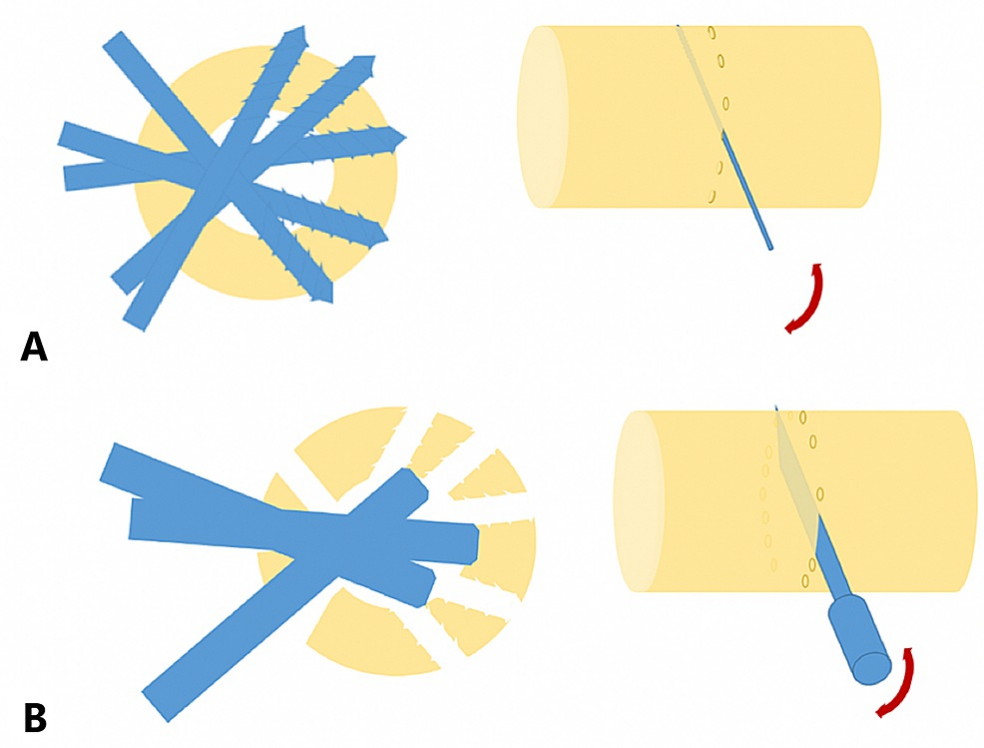
Schematic of the osteotomy. (A) Represents the drilling process for the osteotomy and (B) represents the use of the chisel to complete the procedure. In both, the axial plane is shown first, going from left to right.

The application of a Castaman unilateral external fixator was done under general anesthesia. A tourniquet was used in tibia cases. The osteotomy was performed with a minimally invasive technique. A small skin incision was made and we drilled into the bone at different angles in the same axial plane. Using a chisel, we completed the osteotomy (Figure 1).

**Figure 2:**
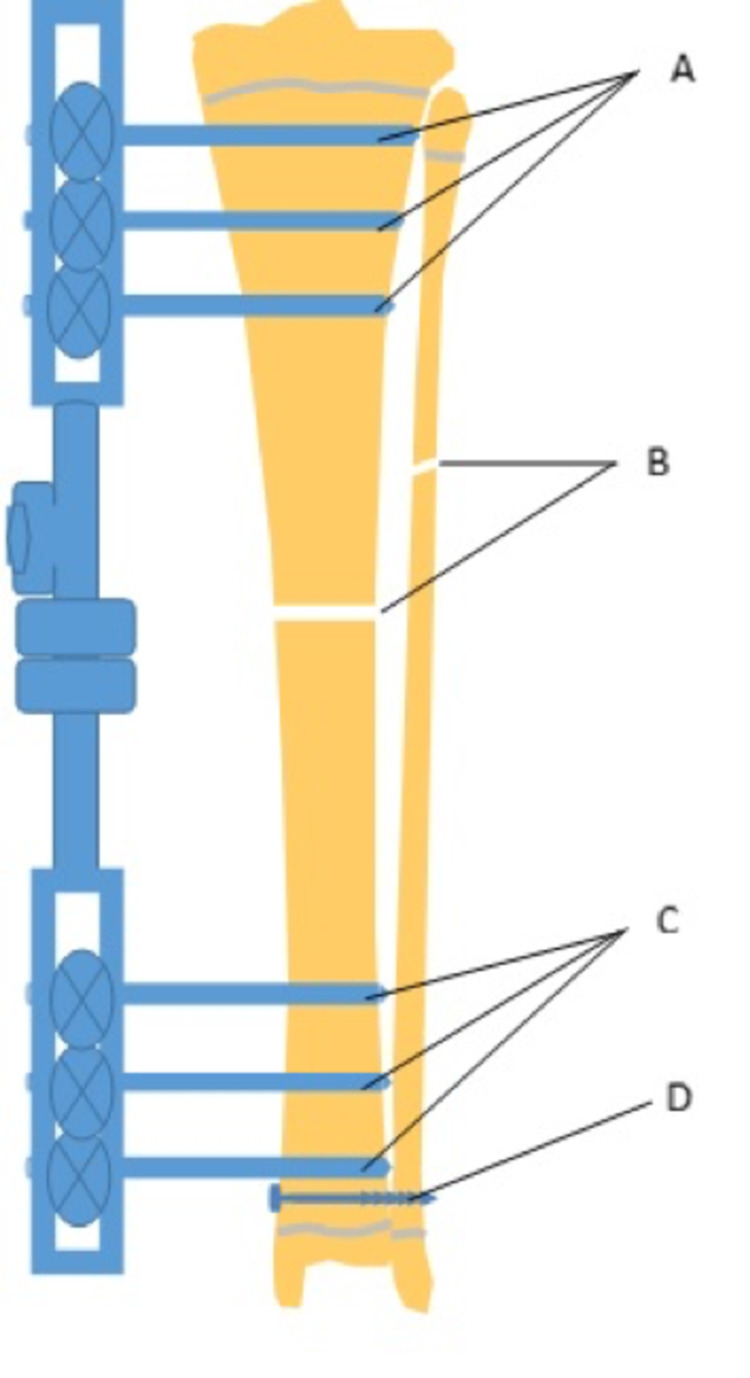
Diagram of an external fixator application after a tibial and fibula osteotomy. (A) Pins inserted proximal to the tibial osteotomy, (B) osteotomy in tibia and fibula, (C) pins inserted distally to the tibia osteotomy, and (D) inter-syndesmosis screw.

**Figure 3:**
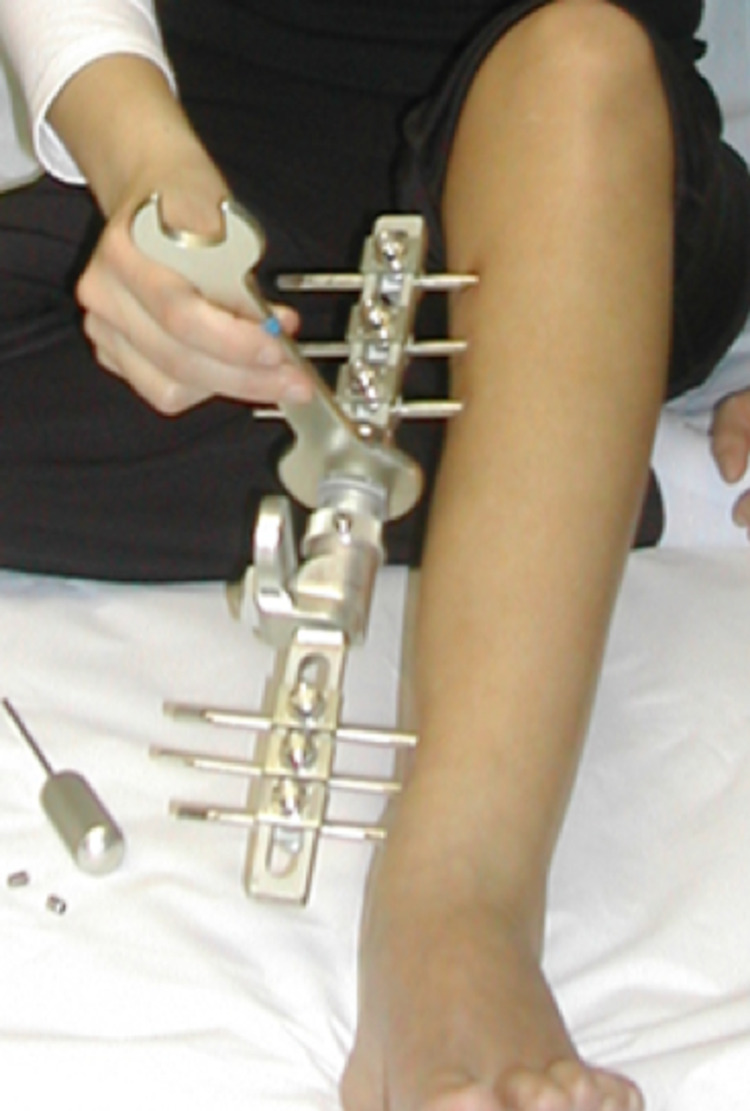
A unilateral external fixator during the lengthening process in the tibia.

Then, proximally and distally, the external fixator pins were inserted and fixed press-fit to the bone. The body of the external fixator was applied at the base of the pins. Cases involving the tibia also had a fibula osteotomy performed and a syndesmosis screw inserted distally (Figures 2-3).

All patients with a congenital LLD (14) underwent a transverse osteotomy followed by the application of a modular external fixator. A week after the osteotomy, we elongated the bone 1 mm per day until it reached the required length.

In all patients with bone loss from trauma, the modular unilateral external fixator was inserted directly and compressed the fracture by shortening the bone. Two weeks after osteotomy, the bone was elongated by 1 mm per day until the desired length was achieved.

All patients with a tibial origin of LLD underwent a tibia osteotomy. A full fibula osteotomy was also performed, with the insertion of a screw fixed distally to the tibia and fibula near the level of their syndesmosis. In order to avoid equinus, a lengthening tenotomy of the Achilles tendon was performed afterwards on all patients.

Families were educated about the external fixator lengthening function. Lengthening every day was performed in collaboration with the patients and their families, or by the patients themselves. Full healing was defined as the patient being able to ambulate without crutches.

Statistical analysis

All continuous variables were analyzed using the student's t-test. Dichotomous variables were analyzed using Fischer’s exact test. The significance level was determined at p<0.05. Statistical analyses were performed with Soscistatistics t-test calculators, Fischer’s exact test calculators, and Graphpad Prism.

## Results

There were 11 females and 8 males, with an average age of 12.7 years (5-25) old. There were 14 cases of LLD in the tibia and 5 in the femur. Three of the five femur cases occurred in the trauma subgroup. There were 14 cases of congenital LLD and 5 cases of traumatic LLD (Table 1). The mean overall LLD was 3.9 cm (2.3-5.2). The mean follow-up until healing was 10 months (5-22).

**Table 1:** Comprehensive patient review. R: right, L: left.

Patient	Gender	Age	Bone	Side (right vs left)	Etiology (congenital vs trauma)	Leg length discrepancy	Months of follow-up until healed
1	F	9	Tibia	R	Congenital	4.2	7
2	F	5	Tibia	L	Congenital	5.2	8
3	M	8	Tibia	R	Congenital	4.8	9
4	F	14	Tibia	R	Congenital	3.7	8
5	M	15	Tibia	R	Congenital	4.2	8
6	F	15	Tibia	L	Congenital	3.6	9
7	F	18	Tibia	R	Congenital	2.5	8
8	M	9	Tibia	L	Congenital	4.1	8
9	F	13	Tibia	R	Congenital	4.5	8
10	M	10	Tibia	L	Congenital	3.8	5
11	M	9	Tibia	R	Congenital	3.8	6
12	F	8	Tibia	L	Congenital	4.7	9
13	M	7	Tibia	R	Congenital	4	7
14	F	6	Femur	R	Congenital	4	9
15	F	7	Femur	L	Congenital	3.1	5
16	M	25	Tibia	L	Trauma	3.5	20
17	F	20	Femur	R	Trauma	3	19
18	M	22	Femur	R	Trauma	2.3	15
19	F	23	Femur	L	Trauma	4.8	22

Sex, bone, sidedness, and leg length discrepancy were not found to be significant (p>0.05). Patients with a congenital LLD were found to be younger than patients with traumatic LLD (10.2 years versus 22.5 years, p=0.000013), more likely to have a discrepancy at the tibia (p=0.034), and had a shorter time frame until full healing (7.6 months versus 19 months, p<0.00001; Table 2).

**Table 2:** Congenital versus trauma etiology comparison. *Statistically significant, p<0.05. R: right, L: left.

	Congenital (15)	Trauma (4)	p-value
Sex	9 female/6 male	2 female/2 male	1
Age (years)	10.2 (5–18)	22.5 (20–25)	0.000013*
Bone	Tibia (13), femur (2)	Tibia (1), femur (3)	0.0374*
R vs L (right vs left)	R (9), L (6)	R (2), L (2)	1
Leg length discrepancy (cm)	4.0 (2.5–5.2)	3.4 (2.3–4.8)	0.1659
Follow-up time till full healing (months)	7.6 (5–9)	19 (15–22)	<0.00001>

Sex, age, sidedness, and leg length discrepancy were not found to be significant (p>0.05). Patients with a tibial LLD were more likely to have a congenital etiology (p=0.0374) as noted in Table 2 and had a shorter time until full healing compared to patients with a femur LLD (8.5 months versus 14 months, p=0.03541) as noted in Table 3.

**Table 3:** Tibia versus femur site comparison. *Statistically significant, p <0.05.

	Tibia (14)	Femur (5)	p-value
Sex	7f/7m	4f/1m	0.3378
Age (years)	(8–25)	(6–23)	0.2511
R vs L	L (8), R (6)	L (2), R (3)	0.6285
Etiology	Congenital (13), trauma (1)	Congenital (2), trauma (3)	0.0374*
Leg length discrepancy (cm)	4.0 (2.5–5.8)	3.4 (2.3–4.8)	0.2113
Follow-up time till full healing (months)	8.5 (6–20)	14 (5–22)	0.03541*

**Figure 4:**
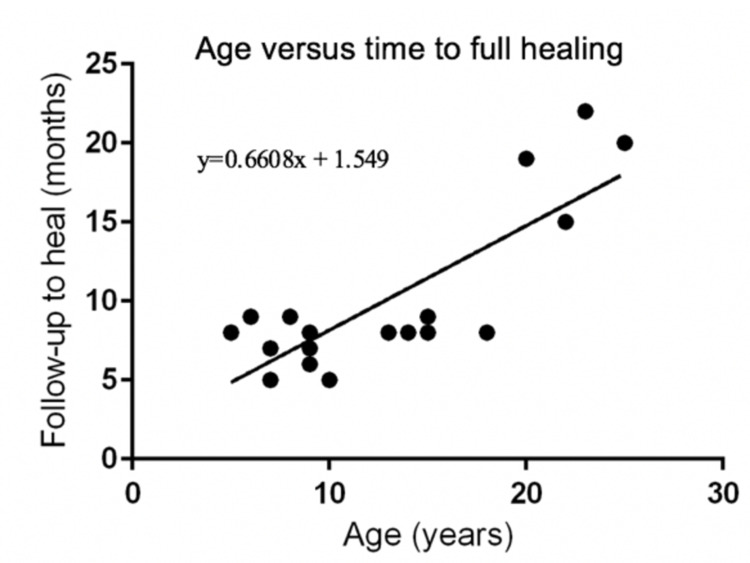
Linear regression analysis of age and time to full healing.

**Figure 5:**
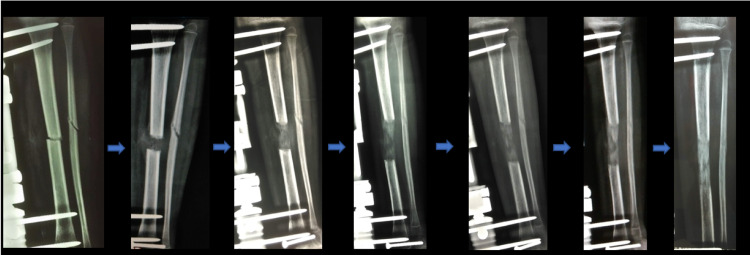
Osteotomy healing and bone lengthening progression of patient 2. From left to right, progressive X-ray images of patient 2 after osteotomy and application of the external fixator. This patient had the largest leg length discrepancy.

Linear regression showed a statistically significant correlation (p<0.0001) between age and follow-up time until full healing (Figure 4). There were no complications. In patient 2 (Figure 5), the anatomy was not appropriate for the standard external fixator size. We inserted the smallest external fixator size in, but the maximum length that this size could achieve was 5 cm instead of the 5.2 cm of discrepancy due to the fixator size. After completing treatment, the patient had a discrepancy of 0.2 cm, but this was not clinically important in her daily activities, and her parents were happy with the result. After eight months of treatment, she walked without crutches and began to run without difficulty. There were no incidents of pseudarthrosis in any patients. All tibial elongation cases required a lengthening tenotomy of the Achilles tendon after the initial tibial osteotomy. In three cases, an equinus position of the foot occurred, which was resolved by Achilles tendon lengthening until full dorsiflexion with splinting in this position for three weeks.

## Discussion

Treatment with a unilateral external fixator was originally developed a long time ago but is still viable. Development of better technology and additional techniques with equivalent or better outcomes, such as the circular external fixator, has resulted in more accurate angular correction with the help of software [10-13]. Despite this, there are still difficulties in getting these technologies to the necessary populations across the globe because of their cost. There are no studies that effectively compare the cost of treatment with external fixators with other modalities, especially as a function of efficacy in different countries.

One main drawback of other fixator systems, which is especially pertinent as health care continues to battle with financial issues, is the cost of the implant, which is considerably higher than unilateral systems. At our hospital, a circular external fixator is more expensive for the patient, with insurance often not covering the cost. In the only cost comparison in the literature, Shore et al. [14] reviewed the cost of pediatric diaphyseal fracture treatment with a circular external fixator versus a uniplanar external fixator and concluded that the cost of a circular external fixator for the index procedure was considerably higher. However, in their cohort, there were complications necessitating a return to the OR, and after compensating for complication costs, they did not find statistical significance. The unilateral external fixator is an economically efficacious device with few or no complications with proper patient selection. Considering the cost component, our more rudimentary techniques show satisfactory results in a resource-limited environment. At our hospital, a circular external fixator is more expensive for most patients, with insurance often not covering the cost.

All of our patients were successfully treated for LLD. The unilateral external fixator can still play an excellent role in LLD in both congenital and traumatic etiologies. Our results showed that traumatic LLD took a longer time to heal, likely due to bone conditions after traumatic events. These patients often have bone loss, so the healing process lasts longer than in congenital LLD. The lengthening procedure started only after the shortening of the limb and callus formation. Femur sites seemed to take longer to heal, but this seems more likely to be due to age again. We had two femur discrepancies due to congenital LLD (ages 6 and 7) and three femur discrepancies due to traumatic LLD (ages 20, 22, and 23).

This study has limitations. Due to the retrospective nature of its design, we were not able to gather patient and parent perspectives on the final treatment outcome. We did not study the associated clinical features and complaints, and with many of the subjects being so young, they were likely unable to properly voice their symptoms. The sample size of the population studied is small; using a larger cohort and including costs would be an ideal future study.

## Conclusions

We conclude that bone lengthening utilizing the unilateral external fixator is a good method and is cost-effective for bone lengthening where more advanced techniques are not available or cost-prohibitive. It is simple, and patients and families can collaborate with the surgeon to get a good final result. Patients are generally satisfied and can ambulate well after healing. In a resource-limited environment with cost as a barrier, if used correctly and judiciously, the unilateral external fixator can yield good results.

## References

[1] Vogt B, Gosheger G, Wirth T, Horn J, Rödl R (2020). Leg length discrepancy- treatment indications and strategies. Dtsch Arztebl Int.

[2] Aronson J (1997). Limb-lengthening, skeletal reconstruction, and bone transport with the Ilizarov method. J Bone Joint Surg Am.

[3] Castaman E, Coppola L, Marenzi R (2014). Fissatori Esterni e Riabilitazione Concetti Integrati tra Chirurgia e Trattamento.

[4] Danzinger MB, Kumar A, DeWeese J (1995). Fractures after femoral lengthening using the Ilizarov method. J Pediatr Orthop.

[5] Docquier PL, Rodriguez D, Mousny M (2008). Three-dimensional correction of complex leg deformities using a software assisted external fixator. Acta Orthop Belg.

[6] Golubović I, Ristić B, Stojiljković P (2016). Results of open tibial fracture treatment using external fixation. Srp Arh Celok Lek.

[7] Dammerer D, Kirschbichler K, Donnan L, Kaufmann G, Krismer M, Biedermann R (2011). Clinical value of the Taylor spatial frame: a comparison with the Ilizarov and Orthofix fixators. J Child Orthop.

[8] Eidelman M, Bialik V, Katzman A (2006). Correction of deformities in children using the Taylor spatial frame. J Pediatr Orthop B.

[9] Giotakis N, Narayan B (2007). Stability with unilateral external fixation in the tibia. Strategies Trauma Limb Reconstr.

[10] Juan JA, Prat J, Vera P (1992). Biomechanical consequences of callus development in Hoffmann, Wagner, Orthofix and Ilizarov external fixators. J Biomech.

[11] Manner HM, Huebl M, Radler C, Ganger R, Petje G, Grill F (2007). Accuracy of complex lower-limb deformity correction with external fixation: a comparison of the Taylor Spatial Frame with the Ilizarov ring fixator. J Child Orthop.

[12] Matsubara H, Tsuchiya H, Sakurakichi K, Watanabe K, Tomita K (2006). Deformity correction and lengthening of lower legs with an external fixator. Int Orthop.

[13] Rozbruch SR, Fragomen AT, Ilizarov S (2006). Correction of tibial deformity with use of the Ilizarov-Taylor spatial frame. J Bone Joint Surg Am.

[14] Shore BJ, DiMauro JP, Spence DD, Miller PE, Glotzbecker MP, Spencer S, Hedequist D (2016). Uniplanar versus Taylor spatial frame external fixation for pediatric diaphyseal tibia fractures: a comparison of cost and complications. J Pediatr Orthop.

